# Quantified assessment of 3D nystagmus in BPPV: practical considerations

**DOI:** 10.3389/fneur.2025.1549407

**Published:** 2025-03-31

**Authors:** Kamran Barin, Michelle R. Petrak, Amy R. Cassidy, Susan L. Whitney

**Affiliations:** ^1^Department of Otolaryngology Head and Neck Surgery, The Ohio State University, Columbus, OH, United States; ^2^Northwest Speech and Hearing, Arlington Heights, IL, United States; ^3^Interacoustics, Middelfart, Denmark; ^4^University of Pittsburgh Medical Center, University of Pittsburgh School of Health and Rehabilitation Sciences, Pittsburgh, PA, United States; ^5^Departments of Physical Therapy and Otolaryngology, University of Pittsburgh, Pittsburgh, PA, United States

**Keywords:** BPPV, torsion, nystagmus, Dix-Hallpike, canaliths, posterior canal

## Abstract

Patients with posterior canal benign paroxysmal positional vertigo (BPPV) have a characteristic response of torsional-vertical nystagmus after a Dix-Hallpike maneuver. The nystagmus usually has a delayed onset with the intensity increasing rapidly and then subsiding over a relatively short duration of less than 1 min. We recorded horizontal, vertical, and torsional eye movements with a VNG system in 15 patients with case histories consistent with BPPV. The nystagmus response patterns were quantified by the latency, peak nystagmus intensity, duration, rise time, and fall time parameters. The results showed a high degree of variability in the response parameters, which signifies that a typical response pattern is not universal in patients with BPPV. In addition to the torsional-vertical nystagmus response, all patients exhibited different levels of horizontal nystagmus. However, the direction and the timing of the responses were not consistent. Some patients showed nystagmus patterns that lasted much longer than 1 min. Other patients had lower levels of nystagmus in response to the Dix-Hallpike contralateral to the affected side. The differences in response patterns may signify differences in the composition or the placement of otoconia within the canal, which may affect the patient symptoms and the outcome of the repositioning maneuvers. However, some of the variability may reflect how the test is performed and how the eye movements are recorded. The purpose of this study was to demonstrate the need for standardization of nystagmus recording protocols because responses to the Dix-Hallpike maneuver are influenced by many factors such as the gaze direction and whether the measurements are made from the ipsilateral or contralateral eye.

## 1 Introduction

Benign paroxysmal positional vertigo (BPPV) is the most prevalent cause of dizziness and vertigo, especially in patients over the age of 50 (1). The majority of the BPPV cases are due to the canalolithiasis of the posterior canal but the estimates for the prevalence of other variants have increased in recent years (2, 3). Patients with typical posterior canal BPPV have a characteristic response after a Dix-Hallpike maneuver consisting of up-beat and torsional nystagmus with the upper pole of the eye beating toward the undermost ear. The nystagmus usually has a short delay with the intensity increasing rapidly and then subsiding over a period of less than 1 min.

Recent evidence suggests that the prevalence of pure posterior canal BPPV may not be as high as once thought (4). Involvement of the other semicircular canals and the placement of the canaliths in atypical areas of the canal are cited as some of the variations of the typical posterior canal BPPV (5). Management of such patients with atypical BPPV has been shown to be more complicated compared to the patients with typical posterior canal BPPV (6).

Measurement of torsional eye movements may provide better insight into the nature of BPPV and the involved canals. The first attempts to quantify torsional eye movements date back to 1980’s and 1990’s [see (7) for a review]. Although three-dimensional (3D) nystagmus recordings in BPPV patients have been reported, few have specified technical issues that may have affected the observed responses (8). Recent developments have provided the capability of using the VNG goggles for measuring 3D eye movements during Dix-Hallpike maneuvers.

The aims of this study were to measure 3D eye movements in patients during the Dix-Hallpike maneuver and to define a series of parameters to summarize nystagmus response patterns. Furthermore, we tested a small number of patients with a history of BPPV to determine how close nystagmus parameters matched the typical response parameters. The aim was not to provide statistical inferences for this small sample. Rather, the aim was to identify the test protocols that may affect the response parameters and must be standardized for routine analysis of 3D eye movements in suspected BPPV patients.

## 2 Materials and methods

### 2.1 Participants

This ongoing study is being conducted at the University of Pittsburgh Medical Center (UPMC). We report the results on 15 patients with case histories consistent with BPPV. Patients with gaze or saccade abnormalities were excluded based on the calibration tracings. Patients included 4 males and 11 females with the age mean/median of 63.2/64 and range of 36–85. They completed a comprehensive history and were evaluated for a history of migraine and anxiety.

Twelve patients underwent Dix-Hallpike maneuvers to both sides. Three patients underwent only one Dix-Hallpike maneuver because of their symptoms. Patients with no torsional nystagmus in at least one of the maneuvers were excluded.

### 2.2 Eye movement recording and analysis

Horizontal, vertical, and torsional eye movements were recorded and analyzed with the VisualEyes^™^ VNG system (Interacoustics, Denmark). Torsional eye movements were quantified based on the method described by Otero-Millan et al. (7). They validated their method by comparing the estimated torsion from video recordings with those recorded by the scleral search coil method. The VNG implementation of the torsion algorithm was further evaluated. The accuracy depends on the gaze direction, but it is always within 1°.

**Figure 1:**
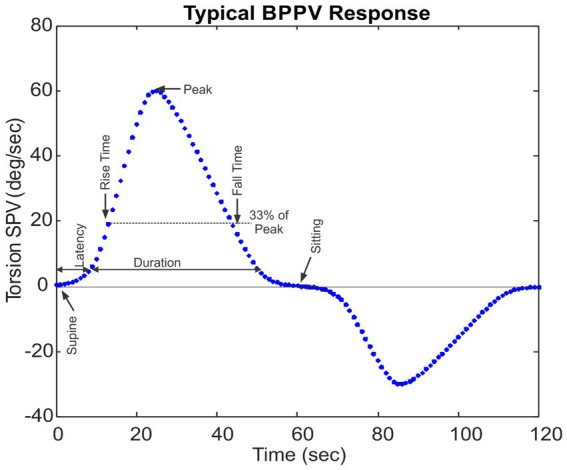
Slow phase velocity (SPV) torsional nystagmus pattern during the Dix-Hallpike maneuver for typical BPPV. The nystagmus reverses when sitting the patient up.

The nystagmus intensity in patients with typical posterior canal BPPV follows a characteristic pattern after a Dix-Hallpike maneuver. Figure 1 shows the typical slow-phase velocity (SPV) pattern for the torsional nystagmus, but the vertical nystagmus also follows a similar pattern. We summarize this pattern using the following parameters:

1. Latency (sec)—The time difference from when the patient is placed in the supine position to the time that the SPV reaches 10% of the peak SPV.
2. Rise time (sec)—The time from the SPV peak to when the response is 33% of the peak SPV before reaching the peak.
3. Fall time (sec)—The time from the SPV peak to when the response declines to 33% of the peak SPV after reaching the peak.
4. Duration (sec)—The time difference from the end of Latency to when the SPV declines to 10% of the peak SPV. The reason for choosing 10% rather than complete disappearance of nystagmus is that it is difficult for nystagmus detection algorithms to identify fast and slow phases of nystagmus when nystagmus intensity is small.
5. Peak (°/s)—The maximum SPV.

We also noted if the nystagmus pattern reversed when the patient was brought up from the supine to the sitting position. This type of reversal enhances the diagnosis of posterior canal BPPV (9).

## 3 Results

Table 1 is a summary of the Dix-Hallpike results. In this patient group, three had torsional nystagmus for the right Dix-Hallpike, 11 for the left Dix-Hallpike, and one for both maneuvers. All patients with positive right-sided responses produced torsion beating toward the undermost ear and up-beat nystagmus. Two patients with positive left-sided responses produced down-beat nystagmus with one torsion beating toward the uppermost ear. The remainder of positive left-sided responses produced torsion beating toward the undermost ear and up-beat nystagmus. For the bilateral case, both sides produced down-beating vertical nystagmus and torsional nystagmus with the upper pole of the eye beating left. Most cases showed reversal of nystagmus on sitting up but in four cases of positive left Dix-Hallpike, the nystagmus did not reverse. In addition to torsional-vertical nystagmus, all patients produced horizontal nystagmus during the Dix-Hallpike maneuver. However, the direction and timing were inconsistent (Table 1). Interestingly, all but five subjects produced smaller nystagmus responses to the unaffected side.

**Table 1:** Summary of right and left Dix-Hallpike responses.

Sub #	Age/sex	Right Dix-Hallpike				Left Dix-Hallpike			
		Torsion (T)	Vertical (V)	Horizontal (H)	Reversal on sitting	Torsion (T)	Vertical (V)	Horizontal (H)	Reversal on sitting
1	52 F	R beat	U beat	L beat	Yes (T/V/H)	—	—	—	—
2	66 M	R beat	U beat	R beat	Yes (T/V)	L beat	U beat	L beat	Yes (V)
3	72 F	R beat	U beat	R beat	Yes (T/V/H)	—	—	—	—
4	68 F	R beat	U beat	R beat	Yes (T/V)	L beat	U beat	L beat	Yes (T/V)
5	62 M	—	U beat	R beat	No	L beat	U beat	L beat	No
6	54 F	R beat	D beat	R beat	Yes (H)	L beat	U beat	R beat	Yes (T/V/H)
7	67 F	—	—	—	—	L beat	U beat	R beat	Yes (T/V/H)
8	36 F	R beat	D beat	R beat	Yes (H)	L beat	U beat	R beat	Yes (T/V/H)
9	57 F	R beat	D beat	L beat	No	L beat	U beat	R beat	Yes (T/V)
10	79 M	R beat	D beat	L beat	No	L beat	U beat	R beat	Yes (T/V/H)
11	61 F	L beat	U beat	—	No	L beat	U beat	R beat	No
12	58 F	R beat	U beat	R beat	No	L beat	U beat	L beat	Yes (T/H)
13	85 F	—	D beat	L beat	No	R beat	D beat	R beat	No
14	67 F	L beat	U beat	R beat	Yes (T/V)	L beat	D beat	R beat	No
15	64 M	L beat	D beat	L beat	No	L beat	D beat	L beat	Yes (V)

Entries with the gray background had positive responses for right Dix-Hallpike whereas entries with the blue background had positive responses for left Dix-Hallpike. One subject had responses to both sides.

**Figure 2:**
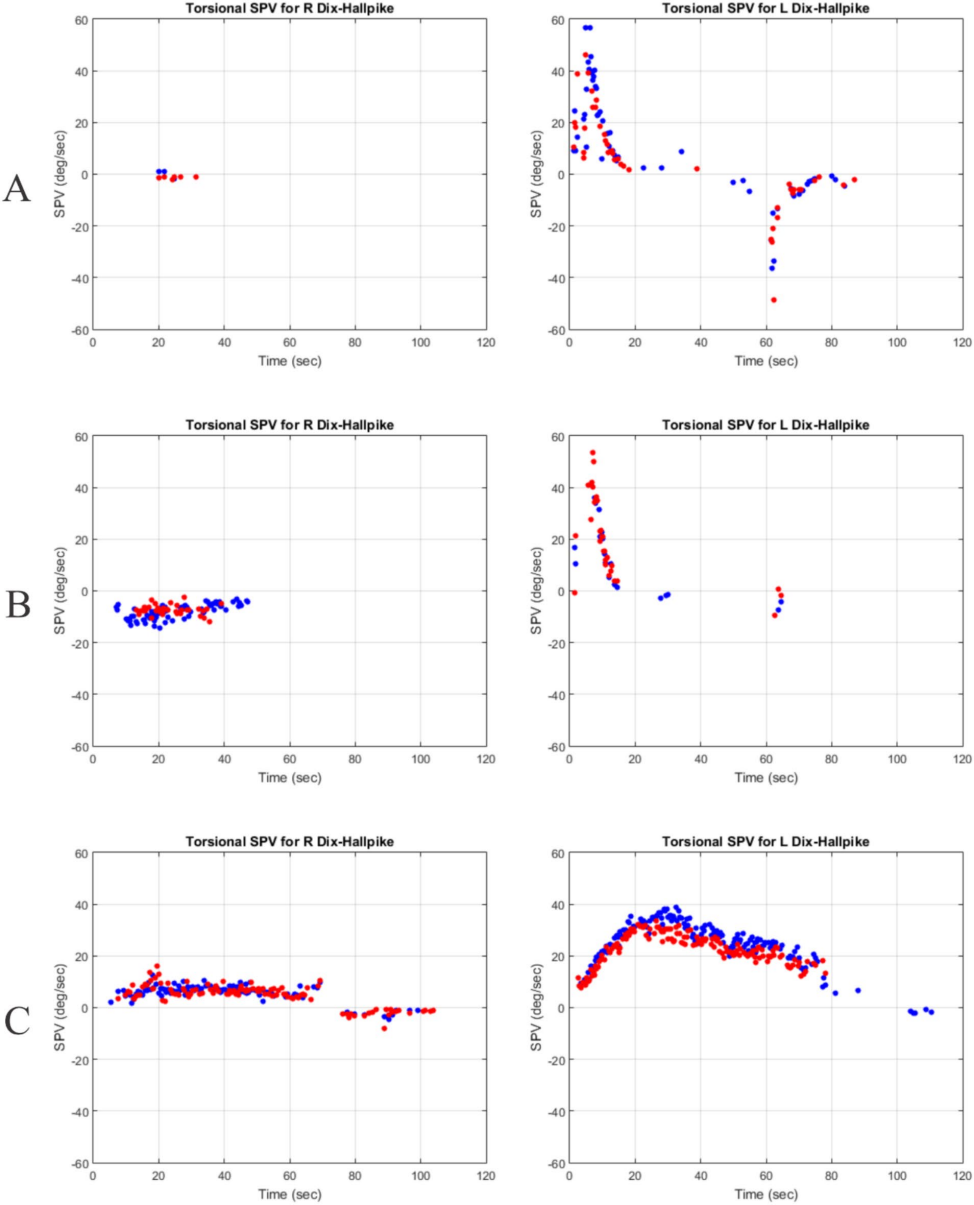
Response patterns. Red dots represent torsional slow phase velocities (SPVs) of the right eye and blue dots represent torsional SPVs of the left eye. **(A)** No response to right Dix-Hallpike and typical response for left Dix-Hallpike. **(B)** Small response to right Dix-Hallpike and typical response for left Dix-Hallpike. **(C)** Long right-beating torsional response present bilaterally in the same direction but much stronger for left Dix-Hallpike.

Figure 2 shows three different torsional nystagmus response patterns. Figure 2A represents a case where the nystagmus pattern follows the typical BPPV pattern for one side and no response to the opposite side. Figure 2B represents a case where the nystagmus pattern follows the typical BPPV pattern for one side and there is a much smaller response to the opposite side. Figure 2C represents a case where the nystagmus is beating in the same direction for both sides and the duration is much longer than the typical response.

**Figure 3:**
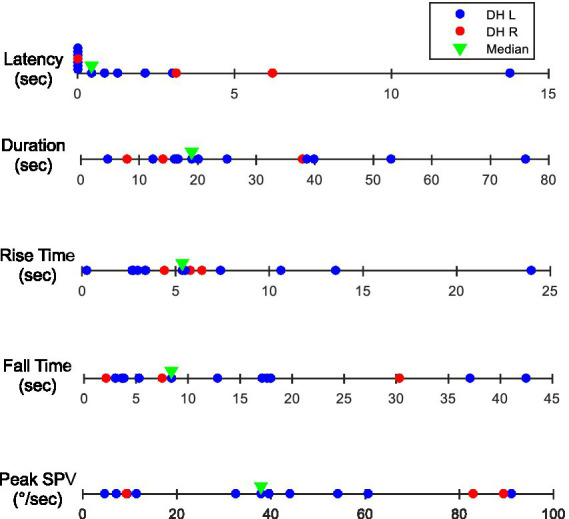
Calculated response parameters for each subject and their median for the positive Dix-Hallpike maneuver (red dots for right Dix-Hallpike, blue dots for left Dix-Hallpike). The median values are identified with a green triangle. DH, Dix-Hallpike; SPV, slow phase velocity.

Figure 3 shows the response parameters calculated for each subject. The median/(range) for each parameter is: latency 0.4/(0.0–13.8) sec, duration 18.9/(4.7–76.0) sec, peak 37.9/(4.8–91.0) deg/sec, rise time 5.4/(0.3–24.0) sec, and fall time 8.4/(2.1–42.5) sec.

## 4 Discussion

As demonstrated in Figure 3, the nystagmus response parameters exhibit a high degree of variability. This variability may be associated with the placement, composition, and size of the canaliths representing variants of BPPV. However, some of the variability could be the result of how the Dix-Hallpike maneuver is performed and how the resulting eye movements are recorded and interpreted. Here we discuss both possible causes and their effects on each nystagmus parameter.

### 4.1 Latency

**Figure 4:**
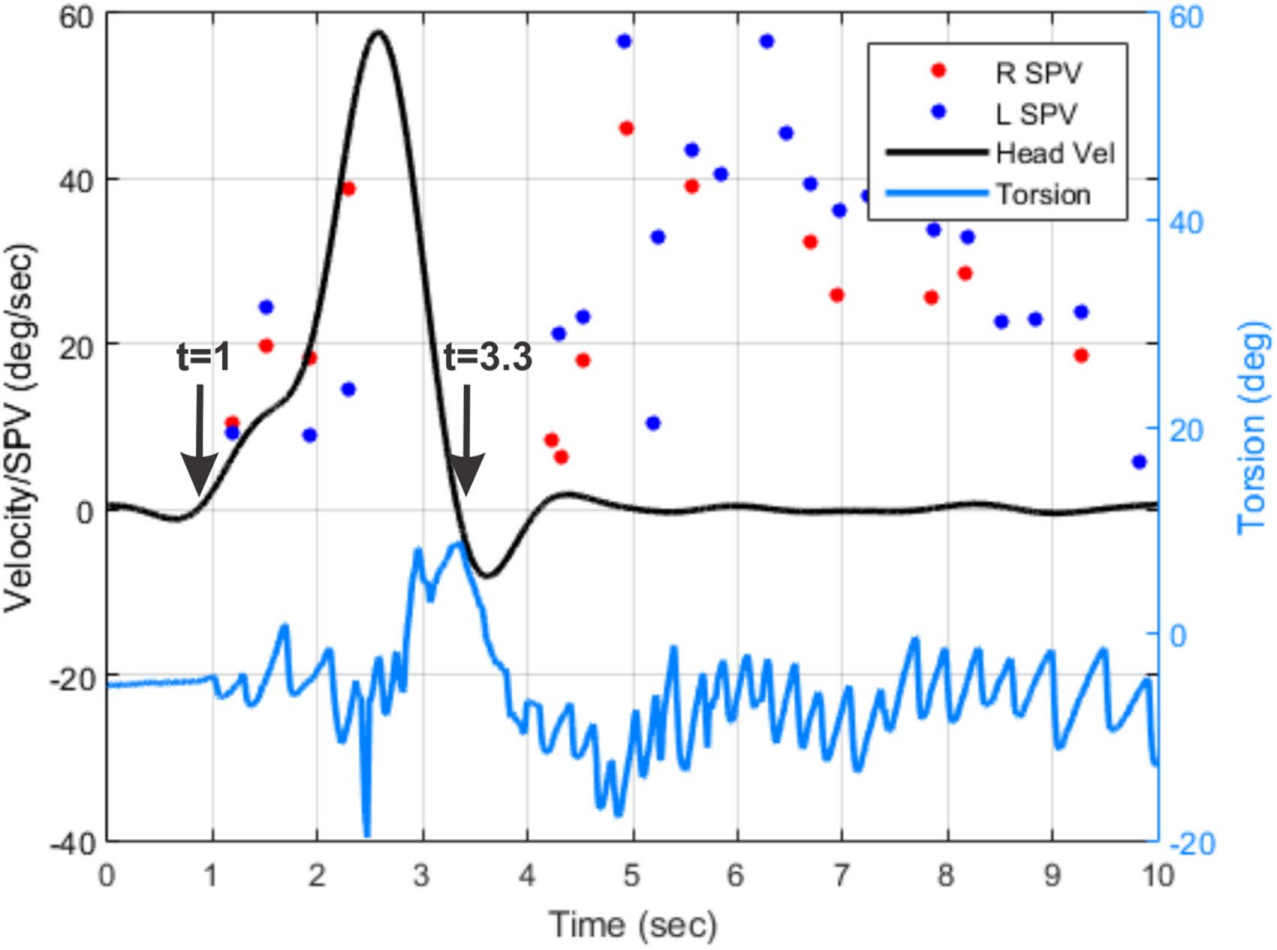
An example of torsional eye position (light blue tracing), torsional SPVs (red and blue dots), and the head velocity (black tracing) for the first 10 s following the left Dix-Hallpike maneuver for one subject.

The response latency in BPPV is usually associated with whether the canaliths are free-floating in the canal or if they are adhering to the cupula (10). In cupulolithiasis, the latency is much shorter than in canalithaisis. The latency can also be affected by the initial position of canaliths as well as their numbers and sizes. However, technical issues can affect the measurement of latency. Figure 4 shows an example of the head velocity tracing along with the torsional nystagmus and SPV patterns following the Dix-Hallpike maneuver. The expected initiation of the head movement is at *t* = 0 s but the actual head movement begins around *t* = 1 s. The nystagmus that occurs during the head movement is likely to be motion-induced nystagmus due to vertical canal stimulations and not related to the movement of the particles. This nystagmus is not taken into consideration when testing without recordings because the examiner does not begin to observe the eye movements until the patient is placed in the supine position. Similarly, for the purpose of quantifying the latency, recorded responses should be standardized to measure the latency only after the head has come to a stop and ignore any nystagmus before that. We identified the starting point for the observation of the response as the point where the head velocity drops to below 10% of its peak value and does not exceed that until the patient is brought back to the sitting position. For this patient, the starting point for the measurement of latency was identified at *t* = 3.3 s.

### 4.2 Duration

The duration of response in BPPV is also associated with the adherence of canaliths to the cupula (11). In cupulolithiasis, the duration is usually longer than 1 min. Another contributing factor to the response duration may be the size of the particles. Richard-Vitton and Viirre (12) proposed that larger particles move faster through the canal while the smaller ones may take much longer. Technical issues do not seem to affect the measurement of duration as long as the latency is identified accurately.

### 4.3 Peak

The peak SPV of the response depends on several factors such as the composition and proximity of the particles to the cupula (13). For example, the response peak and the associated symptoms are expected to be larger when the particles are clumped and closer to the cupula. This association can also explain the well-known phenomenon of response fatigue in some cases when the Dix-Hallpike maneuver is repeated. Previously, the diminished response was thought to be associated with central compensation (13). However, it is more likely that the phenomenon is related to the change in the composition and the location of the particles after the first Dix-Hallpike. In addition, proposals for enhancing the Dix-Hallpike responses, such as loaded Dix-Hallpike maneuvers, are aimed at moving the particles to more favorable positions prior to performing the maneuver (14).

**Figure 5:**
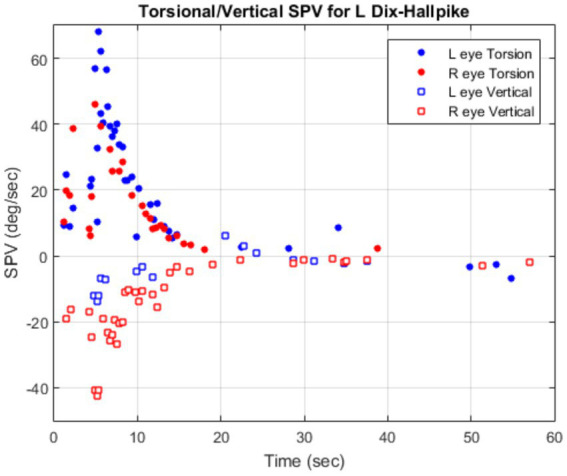
Disconjugate eye movements in BPPV showing greater torsional SPV in the left eye (blue circle) and greater vertical SPV in the right eye (red square) following left Dix-Hallpike.

Technical issues can also affect the measurement of the peak response. It is known that stimulation of vertical canals can cause disconjugate eye movements with higher torsional nystagmus in the ipsilateral eye and higher vertical nystagmus in the contralateral eye (15). Figure 5 demonstrates this phenomenon in one subject following the left Dix-Hallpike maneuver. The peak torsional SPV from the left eye is 62°/sec whereas it is 45°/sec from the right eye. Therefore, one has to standardize which eye is used for the measurement of the peak response. For posterior canal BPPV, the ipsilateral eye for torsional and contralateral eye for vertical eye movements are logical choices. In general, measuring peak responses from both eyes and determining the side with higher response may help with determining the involved canal in BPPV.

Another factor affecting the peak SPV response is the direction of gaze. It is demonstrated that the BPPV nystagmus is primarily torsional when the gaze is directed ipsilateral toward the side of lesion whereas the nystagmus is primarily vertical when the gaze is directed contralateral toward the side of lesion (16). To standardize the procedure, it is best to record and analyze the eye movements with gaze directed straight ahead. This will ensure that both torsional and vertical components of eye movements are present in BPPV patients following the Dix-Hallpike maneuver.

Finally, the head motion velocity during the Dix-Hallpike affects the peak nystagmus responses (17). To address this issue, one can use a head motion sensor to produce relatively consistent head velocities during the Dix-Hallpike maneuvers. Alternatively, the same can be achieved using repositioning chairs.

### 4.4 Rise- and fall-times

Rise- and fall-times, like other nystagmus parameters, depend on the location and composition of the particles. In addition, adherence to the cupula is expected to have an impact on the rise and fall times. For example, canalithaisis is expected to produce short rise and fall times whereas cupulolithiasis will likely generate long rise and fall times (see Figures 2A,C). Cohen and Sangi-Haghpeykar (18) describe cases where patients exhibit symptoms consistent with both canalithaisis and cupulolithiasis. For such cases, rise- and fall-times can provide supporting evidence as the rise-time is expected to be short due to canalithaisis and the fall-time is expected to be long due to cupulolithiasis. The effects of technical issues on rise and fall times are expected to be minimal and as such, they provide more stable parameters for the analysis of nystagmus responses.

### 4.5 Presence of contralateral nystagmus

**Figure 6:**
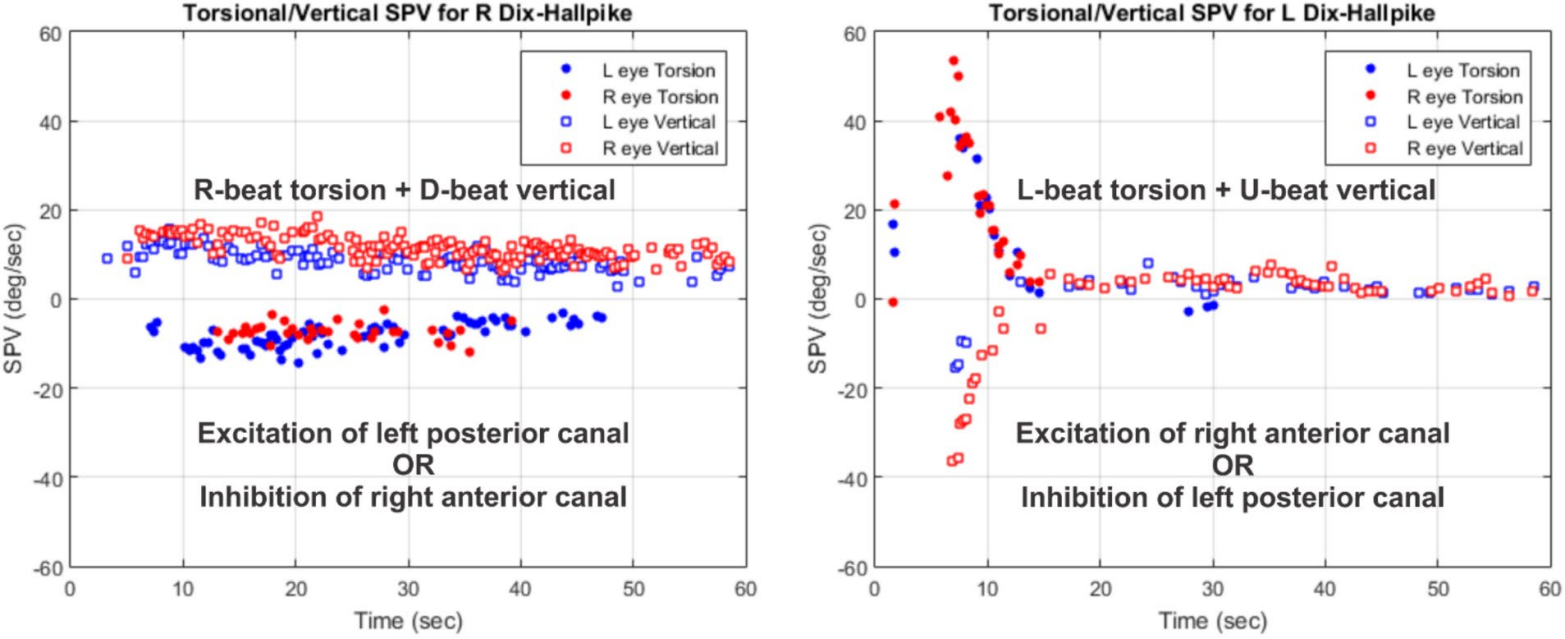
An example of a subject with typical posterior canal responses to one side (left) and smaller responses to the other side (right). The involved vertical canals and whether they are in excitation or inhibition modes are noted based on the nystagmus direction.

Presence of nystagmus in the Dix-Hallpike maneuver to the unaffected side can be considered puzzling for cases of unilateral BPPV. A more careful analysis of nystagmus direction can offer help with the origin of this nystagmus. Table 2 identifies the involved vertical canals and whether they are in excitation or inhibition modes based on the direction of torsional-vertical nystagmus. Figure 6 shows an example of a patient with typical posterior canal BPPV responses for the left Dix-Hallpike maneuver and much smaller responses for the right Dix-Hallpike maneuver. The left Dix-Hallpike responses are most likely due to the canalithaisis of the left posterior canal. For the right Dix-Hallpike, neither the right anterior nor the left posterior canal is in the plane of rotation. It is highly unlikely that the response is due to the excitation of the right anterior canal. Instead, the likely scenario is that the particles in the left posterior canal move up toward the cupula during the right Dix-Hallpike maneuver and generate the inhibitory response. This is possible because the canals are not entirely planar and do not lie exactly in 45°-angle head motion planes. In cases such as this one, once a successful repositioning maneuver has been performed, the contralateral responses also disappear entirely. The above reversal pattern for contralateral nystagmus is not universal. For some cases, inhibition of the involved canal during contralateral maneuvers cannot explain the outcome. For these cases, it is possible that the patients have multi-canal BPPV. Regardless, the present study demonstrates the benefits of recording 3D eye movements because nystagmus patterns are often more complex than what is expected in typical BPPV cases.

**Table 2:** Direction of torsional-vertical nystagmus for the excitation or inhibition of different vertical canals.

Torsion beating…	Vertical beating…	Caused by
Right	Up	Excitation of right posterior canal OR Inhibition of left anterior canal
Left	Up	Excitation of left posterior canal OR Inhibition of right anterior canal
Right	Down	Excitation of right anterior canal OR Inhibition of left posterior canal
Left	Down	Excitation of left anterior canal OR Inhibition of right posterior canal

### 4.6 Presence of horizontal nystagmus

Presence of transient horizontal nystagmus in the absence of torsional-vertical component is an indicator of lateral BPPV, which can be confirmed by the roll test (19). However, there are many instances where the horizontal nystagmus occurs concurrently with strong vertical-torsional nystagmus, which is characteristic of vertical canal BPPV (20). Some of these cases have been considered as multi-canal BPPVs and treated for both vertical and lateral canal involvement (4). In other cases, horizontal nystagmus has been observed in posterior canal BPPV patients without the involvement of lateral canals (8). In our sample, all patients had horizontal nystagmus but the direction and the timing were inconsistent. One possible explanation is that while the labyrinth is a closed structure, individual canals are not. Any endolymph movement in one canal can propagate to other canals through the common crus and generate nystagmus similar to those observed in our sample. This possibility can be verified if horizontal nystagmus diminishes following a successful repositioning maneuver.

### 4.7 Number of abnormal responses for right versus left Dix-Hallpikes

In this study, the number of abnormal responses for the left Dix-Hallpike is much higher than those for the right Dix-Hallpike. This finding seems to contradict the literature (21). However, this discrepancy is difficult to attribute to anything other than the small sample size.

## 5 Conclusion

3D recording of eye movements during Dix-Hallpike maneuvers can help with the identification of BPPV variants and thus lead to better treatment outcomes. The study demonstrates that torsional nystagmus patterns are often more complex than what is expected in typical BPPV cases. Quantification of nystagmus parameters requires standardizing the recording and analysis methods because some aspects of the response may be related to how we perform the test and not to the BPPV variants.

## Data Availability

The raw data supporting the conclusions of this article will be made available by the authors, without undue reservation.
